# Lower extremity mycotic aneurysm in a patient with *Listeria monocytogenes* – associated prosthetic valve endocarditis

**DOI:** 10.1099/jmmcr.0.005095

**Published:** 2017-05-09

**Authors:** Elham Rahmati, P. Jan Geiseler, Rosemary C. She

**Affiliations:** ^1^​ Department of Medicine, Division of Infectious Diseases, Keck School of Medicine of the USC- LAC, Los Angeles, USA; ^2^​ Department of Pathology, Keck School of Medicine of the University of Southern California, Los Angeles, USA

**Keywords:** prosthetic valve endocarditis, mycotic aneurysm, Listeria monocytogenes, intracellular organism, 16S rRNA PCR, Beta-lactam and Aminoglycoside combination

## Abstract

**Introduction.** *Listeria monocytogenes* is a rare etiology of infectious endocarditis with only 30 cases of prosthetic valve and about twice as many native valve infections described in the literature. We describe an unusual presentation of an endovascular embolic phenomenon with associated lower extremity mycotic aneurysm due to *Listeria monocytogenes* prosthetic aortic valve and aortic endograft infection.

**Case presentation.** This is a case of an elderly gentleman with prior history of bioprosthetic aortic valve placement and aortic arch repair who was admitted with several weeks of constitutional symptoms and left lower leg pain. Diagnostic work-up was consistent with thrombosed popliteal artery aneurysm. Blood cultures were positive for *Listeria monocytogenes*. A transesophageal echocardiogram revealed vegetation on the bioprosthetic valve. The patient underwent arterial bypass and ligation of the aneurysm as well as redoing of his aortic valve and aortic graft replacement. Histopathology of the aortic valve was remarkable for acute inflammation and Gram-positive coccobacilli and bacilli occupying intracellular spaces. The results of broad-range bacterial 16S rRNA PCR and sequence analysis of unfixed aortic valve tissue confirmed detection of *L. monocytogenes*.

**Conclusion.** Infective endocarditis attributable to species of the genus *Listeria*is a rare entity. As such, there are no specific guidelines for treatment of *Listeria monocytogenes*endocarditis. However, combination of penicillin or ampicillin with gentamicin is the most acceptable approach described in the literature. Our patient was treated with ampicillin and gentamicin for 6 weeks followed by life-long amoxicillin suppression therapy. The patient remained asymptomatic at a 6 months follow up visit.

## Abbreviation

PCR, Polymerase Chain Reaction.

## Introduction

*Listeria monocytogenes* is a non-spore forming facultatively anaerobic Gram-positive bacillus which is mainly known for its syndromes of gastrointestinal infection, meningitis, meningoencephalitis and bacteremia at extremes of age. This organism is a rarely reported etiology of infectious endocarditis with only 30 cases of prosthetic valve [1–6] and about twice as many of native valve infections in the literature. The first case of *Listeria monocytogenes* native valve endocarditis was described in 1955 and prosthetic valve infection was described in 1976 [7, 8]. Up to 2004, only 23 cases of prosthetic valve infection with *Listeria monocytogenes* had been reported [1, 9] and since then seven additional cases have been added [1–6].

The source of *Listeria monocytogenes* infection can be varied as the organism is known to be ubiquitous in the environment and to have a predilection for certain foods including raw vegetables, cold cut meats and unpasteurized dairy products. In addition, species of the genus *Listeria* can be a part of normal intestinal flora.

## Case report

**Fig. 1.**
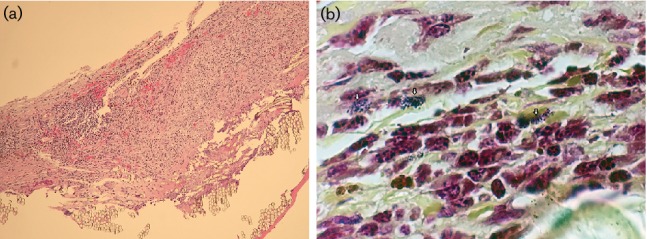
Histopathology of the bioprosthetic aortic valve replaced at surgery shows (a) acute inflammation (Gram stain, ×400 magnification) (b) Gram-stain-positive coccobacilli and bacilli occupying intracellular spaces of occasional mononuclear cells in areas of heavy inflammation (Gram stain, ×1000 magnification).

An 83-year-old gentleman with a history of bioprosthetic aortic valve placement and aortic arch repair for aortic aneurysm two years prior to presentation was admitted with 3–4 weeks history of malaise, chills and left lower leg pain. The patient also had an acute embolic cerebrovascular accident which left him with left sided hemiparesis, left facial droop, dysarthria and mild cognitive impairment six month prior to presentation. On examination, he was afebrile with a blood pressure of 111/70 mm Hg and heart rate of 100 beats per minute. He had a grade II/VI systolic ejection murmur. The left lower extremity was cool to the touch and tender to palpation with diminished pulses. His white blood cell count was 8.7 K µl^−^^1^ (reference range 3.8–10.8 K µl^−1^). A lower extremity angiogram showed a thrombosed popliteal artery aneurysm. He underwent arterial bypass and ligation of the aneurysm on hospital day 3. Both of two blood culture sets from the day of admission were positive for Gram-positive rods which were initially identified by multiplexed PCR (FilmArray BC-ID, bioMerieux) as *Listeria monocytogenes.* Subsequent growth on solid media recovered both *L. monocytogenes*, later confirmed by the public health laboratory, and *Listeria innocua.* No recent history of antibiotics use was reported prior to admission and the patient did not receive any antibiotics upon admission. On hospital day 1, two out of two blood culture sets again were positive for *Listeria monocytogenes* and *Listeria innocua.* From one of the two blood culture sets *Escherichia coli* was also simultaneously isolated. The patient was started on ampicillin and gentamicin, targeting all recovered organisms. Subsequent blood cultures on days 3 and 4 of hospitalization were negative. A transesophageal echocardiogram revealed a vegetation measuring 14×8 mm on the aortic aspect of the bioprosthetic valve. Magnetic resonance imaging of the brain showed a preexisting infarct and multiple new punctate, subacute, locunar infarcts involving the left thalamus and left precentral gyrus. Results of imaging of chest, abdomen and pelvis were remarkable for evidence of splenic infarcts but otherwise no other infectious sources were identified. The patient subsequently underwent a redo of the aortic valve and aortic graft replacement two weeks into admission. Gross pathology of the bioprosthetic valve showed a markedly disrupted trileaflet valve. Histopathology was remarkable for acute inflammation (Fig. 1a) and tissue Gram staining detected Gram-positive coccobacilli in intracellular spaces (Fig. 1b). No Gram-negative bacilli indicative of *E. coli* were observed. The unfixed valve tissue which was sent to a reference laboratory (University of Washington) for broad-range bacterial 16S rRNA PCR and DNA sequence analysis by Sanger sequencing detected *Listeria monocytogenes*.

## Diagnosis

*Listeria monocytogenes* prosthetic valve endocarditis.

## Discussion

Visualization of *L. monocytogenes* in cardiac valve tissues is not well described as the cases of endocarditis reported thus far were either negative for organisms on histopathology, or the histopathological features were not mentioned. We found that, as consistent with gastrointestinal and central nervous system listeriosis, the pathogen was in fact found in mononuclear cells within the inflammatory infiltrate and appeared to be bacillary or coccobacillary (Fig. 1b). Although these findings are not specific to *L. monocytogenes*, it is well known as a facultative intracellular pathogen capable of inducing internalization by host cells. Once internalized, it can escape from host phagolysosomes and initiate cytoskeletal changes in order to spread from cell to cell, thus facilitating invasive entry through the gut epithelium [10]. We were able to observe the presence of the organism in the valve tissue and confirm its identity as *L. monocytogenes* by 16S rRNA gene sequencing which, to the best of our knowledge, has never been reported before. Based only on the Gram staining of resected tissue, the observed organism forms could also resemble streptococcal, enterococcal or diphtheroid organisms. In this case, due to the polymicrobial nature of the blood culture, we felt it was important to pursue definitive identification of the organism by PCR and sequence analysis [11]. However, because Sanger sequencing results yielded *L. monocytogenes,* it probably represented the sole or dominant bacterial organism in the tissue sample. The role played by *L. innocua,* also isolated from multiple blood cultures, is probably minor if any, and there are only rare reports of it causing human infections [12].

Our case was also unusual in that the patient initially presented with acute limb ischemia secondary to a mycotic aneurysm caused by *L. monocytogenes.* He also suffered an ischemic cerebral infarct six months prior to admission which, although too long of duration for subacute endocarditis, such could not completely be excluded as no blood cultures were obtained during that admission. A review of *Listeria monocytogenes* prosthetic valve endocarditis cases published in the literature disclosed only six other cases associated with emboli which were all in patients with prosthetic mechanical valves: one with emboli to coronary arteries [8], three with emboli to the central nervous system [4, 13, 14], and two with emboli to the lower extremities [15, 16]. It has been suggested that peripheral emboli, presence of mechanical rather than biological prosthetic valves and penicillin monotherapy are independent risk factors for mortality among these patients [9].

Our patient did report regular ingestion of foods known to be associated with *Listeria* infections, namely unpasteurized goat milk, feta cheese and cold cut meats from a European deli counter which were presumptively the source of his infection. To the best of our knowledge, no epidemiologically linked *Listeria* food-borne cases were reported in the state of California around the time of this case presentation. It should be noted that source of infection was also unknown in majority of cases of *L. monocytogenes* endocarditis reported in the literature [1]. Upon further questioning, our patient did report some subjective fevers, chills and a vague history of generalized abdominal cramps over the past two week prior to presentation but no other enteric symptoms such as diarrhea were reported. Although a direct association between *Listeria* bacteremia and gastrointestinal cancer has not been established, Fernandez *et al*. reported that seven out of 56 cases of *Listeria* endocarditis had solid tumour cancers, of which four were colonic adenocarcinoma [1]. The initial polymicrobial bacteremia with intestinal organisms in our case made this an even more likely possibility and therefore there was a clinical consideration for colonoscopy. However, our patient had already had a recent normal colonoscopy which was performed at another facility.

In conclusion, infective endocarditis due to species of the genus *Listeria* is a rare entity. As such, there are no specific guidelines for treatment of *Listeria monocytogenes* endocarditis. A review of the literature indicates that combination of penicillin or ampicillin with gentamicin for 4–8 weeks with longer duration for prosthetic valves is the most acceptable approach. The case presented by Makaryus *et al*. was also treated with life-long amoxicillin for prophylaxis immediately after finishing a course of intravenous antibiotics with good response at 18 month follow-up [6]. We also treated our patient with ampicillin and gentamicin for a total duration of 6 weeks. Because there was a small portion of old aortic graft remaining for surgical re-anastomosis, there was concern for potential recurrence of *Listeria* infection and we elected to place our patient on life-long amoxicillin suppression therapy. The patient remained asymptomatic at a 6 months follow up visit.
